# Divergence of discrete- versus continuous-time calculations of the temperature dependence of maximum population growth rate

**DOI:** 10.21203/rs.3.rs-5361425/v1

**Published:** 2024-11-14

**Authors:** Paul J. Huxley, Leah R. Johnson, Lauren J. Cator, Samraat Pawar

**Affiliations:** 1. Department of Statistics, Virginia Tech, Blacksburg, Virginia, USA; 2. Department of Life Sciences, Imperial College London, Silwood Park Campus, Ascot, UK

## Abstract

The temperature dependence of population fitness $\left(r_{\text{m}}\right)$ is key to predicting ectotherm responses to climatic change. Discrete-time matrix projection models (MPMs) are used to calculate $r_{\text{m}}$ because they capture variation in its underlying life-history trait values and time delays inherent in those traits. However, MPM calculations can be laborious and do not capture time’s continuous nature. More complex approaches for calculating temperature-dependent $r_{\text{m}}$ may be more accurate but they are notoriously difficult to parameterise. Ordinary differential equation-based models (ODEMs) offer a relatively tractable alternative of intermediate complexity, but it is unknown whether they broadly agree with MPM calculations when environmental variation is introduced. Here we investigate differences in the predicted temperature dependence of $r_{\text{m}}$ obtained from an ODEM with those calculated from MPMs using temperature- and resource dependent life-history trait data for the disease vector, *Aedes aegypti*. We show that the level of agreement between discrete- and continuous-time representations of temperature-dependent $r_{\text{m}}$ can vary with resource availability and is extremely sensitive to juvenile survival characterisations. This finding suggests that ODEMs can only provide comparable $r_{\text{m}}$ predictions to standard methods when resources are not limiting, questioning the ability of existing mathematical models to reliably predict arthropod responses to environmental variation.

## Introduction

The distribution and abundance of ectotherm populations is strongly shaped by two key components of their ecological niche: environmental temperature and resource availability. Climate change is predicted to have severe impacts on ectotherms through changes in both (Høye et al., 2021; Lawlor et al., 2024). In particular, how these factors affect arthropod populations, the most abundant group of animals on earth (Bar-On et al., 2018), is of significant concern for ecosystem functioning, agriculture, and human health (Costanza et al., 1997; Outhwaite et al., 2022; Pecl et al., 2017; Ryan et al., 2021; Sánchez-Bayo and Wyckhuys, 2019; Skendžić et al., 2021). For example, recent surges in sustained local dengue transmission across Europe have been partially attributed to how ongoing seasonal temperature changes have increased the region’s thermal suitability for *Aedes* mosquitoes (Branda et al., 2023; Nakase et al., 2023; Oliveira et al., 2021).

To understand how variation in environmental temperature and resource availability can influence arthro-pods, we need their thermal performance curves (TPCs) for maximal population growth rate $\left(r_{\text{m}}\right)$—a population’s inherent capacity to grow in the absence of density-dependent factors—a fundamental measure of population fitness (Amarasekare and Savage, 2012; Birch, 1948; Cole, 1954; Huey and Berrigan, 2001; Pawar et al., 2024). Previous efforts have used four different approaches for calculating temperature-dependent $r_{\text{m}}$: matrix projection models (MPMs; Caswell, 1989), ordinary differential equation based models (ODEMs; e.g., Winsor, 1932), delay-differential equation based models (DDEMs; e.g., Amarasekare and Coutinho, 2013; Brass et al., 2021), and Integral Projection models (IPMs; Ellner and Rees, 2006).

Among these methods, DDEMs and IPMs may provide more accurate calculations of $r_{\text{m}}$ than MPMs and ODEMs but they are the most challenging to use—they are notoriously difficult to parameterise and solve, with IPMs, in addition, being computationally costly. Here we focus on discrete-time MPMs and continuous-time ODEM-based approaches for calculating the TPCs of $r_{\text{m}}$ because arthropod data are often not at the required resolution for use in these more complex models. For example, IPMs require at least one continuous trait measurement such as age or body size. This parameterisation challenge is amplified when the goal is to calculate fitness across environmental gradients. In contrast, MPMs and ODEM-based approaches can work well with relatively limited data; for example, they can still provide powerful insights into population dynamics even if only stage-specific vital rates (e.g., development, survival and fecundity) are known (Huxley et al., 2021, 2022; Pawar et al., 2024).

For these reasons, MPMs and ODEM-based approaches are commonly used to calculate $r_{\text{m}}$ in organisms with complex life histories, especially when the goal is to incorporate how different life history stages respond to environmental factors such as temperature. For example, MPMs have been used recently to show that variation in resource availability can have profound effects on the temperature dependence of $r_{\text{m}}$ in the mosquito vector, *Aedes aegypti* (Huxley et al., 2021, 2022).

MPMs can also capture time-delays in life history cycles. For example, matrix columns can be added to incorporate delays introduced by intermittent resource limitation. Despite these strengths, building MPMs to calculate $r_{\text{m}}$ across environmental gradients can be laborious and, because they operate in discrete time steps, they typically do not adequately capture the continuous variation in trait values over time. ODE-based models provide a relatively tractable method to calculate $r_{\text{m}}$ when life history data are incomplete and overcome some of the challenges involved with DDEM and IPM parametrisation. And relative to MPMs, they are less laborious to construct relative and do not suffer from the time-discretization issue. However, the consistency of $r_{\text{m}}$ calculations based on ODEMs across environmental gradients compared to MPMs is largely unknown.

Here, we study the level of agreement between three approaches to calculate the temperature dependence of $r_{\text{m}}$ under variation in resource availability in the disease vector, *Aedes aegypti*: an analytic approximation to $r_{\text{m}}$ derived from an ODE-based model, an explicit MPM simulation, and an analytic approximation to the latter. Our main goal is to evaluate the extent to which $r_{\text{m}}$ calculated from the continuous time stage-structured population model (the analytic $r_{\text{m}}$ model; herein the AE-L model) matches that calculated from the MPM across environmental gradients.

We expected any differences in results between these approaches to stem from how differently they weigh the contributions of life-history traits. MPMs and its E-L-based $r_{\text{m}}$ approximation consider discrete stages and time steps, while continuous-time models and their E-L-based $r_{\text{m}}$ approximation integrate over life stages and time for juvenile mortality and development. For example, Cator et al. (2020) simplified the AE-L model by assuming a fixed mortality rate (an exponentially-declining survivorship curve), which can be integrated analytically to obtain total cumulative juvenile-stage mortality. In contrast, the MPM and its E-L-based $r_{\text{m}}$ approximation allow for more varied juvenile survival patterns, which in turn affects predicted $r_{\text{m}}$. We hypothesize that the AE-L model’s survivorship assumption is valid unless juvenile mortality is high, because, as shown by Cator et al. (2020), $r_{\text{m}}$ is less sensitive to mortality than to development time and fecundity based on a standard stage-structured ODEM.

We show that the AE-L $r_{\text{m}}$ model consistently yields similar temperature- and resource-dependent $r_{\text{m}}$ calculations to the discrete-time E-L equation. However, MPM-derived $r_{\text{m}}$ calculations can diverge from these approaches due to their sensitivity to how juvenile survival trajectories can be shaped by variation in resource availability.

## Materials and Methods

We tested our hypothesis by modelling juvenile mortality in the E-L equation and MPM as either a fixed reduction per time step (“exponential decay”) or as survival until a cut at the transition point (“transitional decay”). The rationale for this approach is based on how the AE-L model effectively assumes that using an exponentially-decaying survival function to integrate over time steps is reasonable because, in the end, it should still approximate the total proportional mortality at the juvenile-to-adult transition point. In contrast, the E-L equation and MPM can have arbitrary patterns allowing for testing of how different characterisations of juvenile survival may influence $r_{\text{m}}$. However, in spite of such flexibility, juvenile mortality is rarely directly measured for many arthropods, which forces practitioners to make strong assumptions (e.g., exponential or transitional decay) about it’s dependence on time and environmental variation.

### *Calculating* $r_{\text{m}}$ *using the MPM*

We use the standard stage-structured MPM (Caswell, 1989) for population change over discrete time steps:

(1) $${\text{N}}_{\text{t}+1}={\text{MN}}_{\text{t}}.$$

Here, ${\text{N}}_{\text{t}}$ is the vector of abundances in the life stage classes (larval, pupal, and adult) at time t and M is the transition matrix. The first row of M is populated by stage specific daily fecundity (the number of female offspring produced per female at stage/age i). The sub-diagonal of M is populated with the probabilities of survival from stage i to stage i+1. Multiplying the transition matrix, M, and stage-structured population size vector, ${\text{N}}_{\text{t}}$, sequentially across time intervals yields the stage-structured population dynamics. Once the stable age/stage distribution (SAD) is reached, the dominant eigenvalue of the system is the finite population rate of increase (λ) (Caswell, 1989). A population reaches SAD when the relative proportion of individuals in each life stage has stabilised (that is, the proportion of the population in each age-stage class remains constant over time), even when the total population size is changing (Caswell, 1989). Then, the intrinsic rate of population growth is $r_{\text{m}}=\text{log}(\lambda )$ (i.e., *per capita* change in the population per unit time).

#### Approximating $r_{\text{m}}$ from the MPM

Assuming SAD, the discrete-time Euler-Lotka approximation (Lotka, 1907) of the above MPM (Eqn 1) is,

(2) $$\sum_{x=1}^{n}e^{-r_{\text{m}}x}l_{x}b_{x}dx=1,$$

Here, $l_{x}$ is age/stage-specific survivorship (probability that an individual survives to age/stage x), and $b_{x}$ is stage-specific fecundity (zero for all juvenile stages). This E-L equation describes the expected lifetime reproductive success of a new-born individual in a stage-structured population growing at the rate $r_{\text{m}}$ once it has reached SAD. To approximate $r_{\text{m}}$ in Eqn 2, we obtain its root through numerical solution, which requires life tables comprised of $l_{x}$ and $b_{x}$ arranged into rows representing age that, in our case, increases daily.

### *Calculating* $r_{\text{m}}$ *from the continuous-time stage-structured population growth model*

Analogous to Eqn. 2, under the same assumption of SAD, the E-L equation for the continuous time (ODE-based) model for a age-structured population change is:

(3) $$\int_{\alpha }^{\infty }e^{-r_{\text{m}}}l_{x}b_{x}dx=1.$$

As in Eqn 2, here $r_{\text{m}}$ is the maximal growth rate once the population has converged on its SAD. However, the life-history traits here are defined slightly differently: $l_{x}$ and $b_{x}$ are age-(not stage-) specific survivorship and fecundity, and we now we have a single parameter α representing the age of first reproduction corresponding to the development time from egg (or neonate) to reproductive adult. Thus, in this formulation we ignore the discreteness of life stages, a simplification that allows us to derive a closed-form analytic approximation for $r_{\text{m}}$ (Cator et al., 2020):

(4) $$r_{\text{m}}\approx \frac{(\kappa +z)\left(\text{log}\left(\frac{b_{\text{max}}}{\kappa +z}\right)-{\alpha z}_{\text{J}}\right)}{\alpha (\kappa +z)+1}.$$

Here, α is egg-to-adult development time (days), $b_{\text{max}}$ is peak reproductive rate (individuals (eggs) × individuals (females) × day^–1^), κ is the fecundity loss schedule (individual^–1^ day^–1^), and zJ and z are juvenile and adult mortality rates (individual^–1^ day^–1^), respectively. Although Eqn 4 is an approximation, it can be shown to be sufficiently accurate as long as $r_{\text{m}}$ is less than 1 (in units of day^–1^; Cator et al., 2020), which is typically true for insect growth rates (Frazier et al., 2006; Pawar et al., 2024). Equation 4 explicitly incorporates the traits underlying $r_{\text{m}}$ (through $l_{x}$ and $b_{x}$ in Eqn 3), so it can be used to analytically understand how variation in these traits propagates through the system to affect $r_{\text{m}}$ (Cator et al., 2020). Note that because Eqn 4 is derived from Eqn 3, it is only valid when the population is at SAD, similarly to the MPM and its analytic approximation (Eqn 2).

### Data

To determine the level of agreement between the AE-L model (Eqn 4), the E-L equation and the MPM across environmental gradients, we used two datasets that describe how resource availability can affect $r_{\text{m}}$ in the disease vector, *Aedes aegypti*. Both datasets are derived from laboratory experiments on this species that were conducted by Huxley et al. (2021; 2022). Huxley et al. (2022) measured the effect of larval competition on the temperature dependence of juvenile development time and mortality rate; α and zJ in Eqn 4, respectively) and adult fitness traits (fecundity rate and mortality rate; $b_{\text{max}}$ and z in Eqn 4, respectively) at five constant temperatures (22, 26, 32, 34 and 36°C) and four resource concentration levels (0.183, 0.367, 0.550, and 0.733 mg ml^–1^). Huxley et al. (2021) measured the effect of variable resource supply on the temperature dependence of the same fitness traits at three constant temperatures (22, 26, 32°C) and two resource supply levels (0.1 and 1 mg larva^–1^ day^–1^).

### *Parametrisation of the* $r_{\text{m}}$ *models*

We parameterised the three $r_{\text{m}}$ models (Eqns 2, 3, 4) with mean trait responses (development time and adult longevity were rounded to zero decimal places) that we calculated using the raw replicate-level data (n=3 replicates at each treatment level; Tables SI1, SI5) from Huxley et al. (2021; 2022). For both datasets, we obtained $z_{\text{J}}$ by dividing the proportion of juveniles that did not survive to adulthood by α. For z, we inverted adult longevity (i.e., z=1/longevity), and, as κ has been shown to only make a very small contribution to $r_{\text{m}}$ (Cator et al., 2020), we assumed that $b_{\text{max}}$ declined with age at a constant rate of 0.01 individual^–1^ day^–1^.

For both datasets, we calculated $r_{\text{m}}$ for every treatment’s replicates by parametrising the $r_{\text{m}}$ models with the trait values shown in tables SI1 and SI5. To calculate $r_{\text{m}}$ with the AE-L model, these values were used directly for each experimental temperature. Due to structural differences between discrete- and continuous-time models, it was necessary to transform these values to estimate $r_{\text{m}}$ using the E-L equation and the MPM.

Calculating $r_{\text{m}}$ using the E-L equation requires life tables comprising of rows populated with $l_{x}$ (the probability that an individual survives to stage x) and $b_{x}$ (stage-specific fecundity; set to zero for all juvenile stages). The total number of rows in each life table was equal to the sum of development time plus adult longevity. At maturity, $b_{x}$ was equal to $b_{\text{max}}$, which decreased at a rate of κ per time step $(b_{\text{max}}-(0.01\times \text{day})$ until death, i.e., the last row of the life table). When juvenile survival probability $\left(l_{x}\right)$ was assumed to decrease at a fixed rate per day, this quantity was obtained by subtracting $z_{\text{J}}\times$ developmental day from 1. When juvenile survival only decreased at the transition point to adulthood, life table rows were populated with 1 until this point. At transition, juvenile survival was obtained by subtracting $z_{\text{J}}\times$ total development time (i.e., α) from 1. Adult survival decreased at a fixed rate per day in all life tables. This quantity was obtained by subtracting 1/adult longevity × day from the juvenile survival probability at transition until the final adult age class (i.e., row).

To estimate $r_{\text{m}}$ using the MPM, each column in the transition matrix (M in Eqn 1) represented one day. The total number of columns in M was equal to the sum of development time plus adult longevity. Reproduction does not occur in the juvenile stages, so the first row of M was populated with zeros until maturity. At maturity, the first row of M was populated with $b_{\text{max}}$ which decreased at a rate of κ per time step (i.e., $b_{\text{max}}-(0.01\times \text{day}$)) until death. The sub-diagonal of M was populated with the probabilities of survival from stage t to stage t+1. As with the life tables for the E-L equation, juvenile survival M was obtained by subtracting $z_{\text{J}}\times \text{day}$ from 1. When juvenile survival decreased at the transition point to adulthood, the sub-diagonal was populated with 1 until this point. At transition, juvenile survival was obtained by subtracting $z_{\text{J}}\times \text{total}$ development time (i.e., α) from 1. Adult survival was obtained by subtracting 1/adult longevity × day from the juvenile survival probability at transition until the final adult age class. The projection matrices were built and analysed in R (R Core Team, 2023) using the popbio package (Stubben and Milligan, 2007). To estimate $r_{\text{m}}$ for the E-L equation, we used the uniroot function in base R (R Core Team, 2023).

### *Fitting Thermal Performance Curves (TPCs) to the* $r_{\text{m}}$ *calculations*

The temperature at which $r_{\text{m}}$ peaks $\left(r_{\text{m}}\;T_{\text{opt}}\right)$ and the value of $r_{\text{m}}$ at its peak $\left(r_{\text{opt}}\right)$ are important parameters for understanding how arthropod populations will respond to long-term sustained climatic warming (Pawar et al., 2024). To predict $r_{\text{m}}\;T_{\text{opt}}$ and $r_{\text{opt}}$, we generated continuous $r_{\text{m}}$ TPCs using non-linear least squares (NLS) in the rTPC pipeline (Padfield et al., 2021). We fitted the Sharpe-Schoolfield TPC model (Kontopoulos et al., 2018; Schoolfield et al., 1981; Eqn SI1) to the replicate-level $r_{\text{m}}$ calculations for each experimental temperature in the larval competition dataset (Huxley et al., 2022). We used this model because it has been theoretically and empirically validated. However, as is the case with most TPC models, the Sharpe-Schoolfield model (Schoolfield et al., 1981) can only be used to predict non-negative responses, so for treatments comprising of both non-negative and negative $r_{\text{m}}$ calculations, we fitted a generalised additive model (GAM) using the mgcv package (Wood and Wood, 2015; Wood, 2011).

The resource limitation dataset (Huxley et al., 2021) does not cover a sufficient range of temperatures to fit continuous $r_{\text{m}}$ TPCs, so we parametrised Eqn 4 with the data for each temperature × resource supply treatment in that study.

## Results

### *Comparison of the* $r_{\text{m}}$ *calculations using the larval competition dataset*

**Figure 1:**
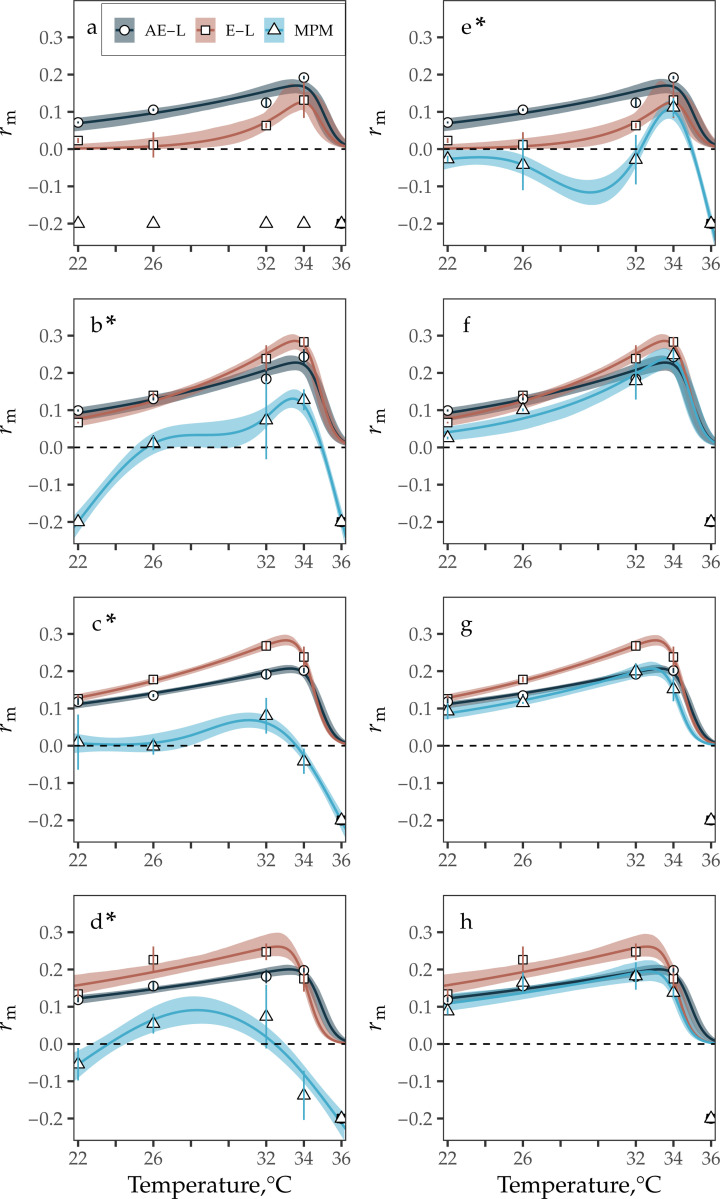
TPCs fitted to $r_{\text{m}}$ calculations from the analytic $r_{\text{m}}$ model (AE-L, grey), the Euler-Lotka equation (E-L, red) and the MPM (blue) for the resource competition dataset (Huxley et al., 2022). (**a – d**) $r_{\text{m}}$ TPCs for the three $r_{\text{m}}$ models across resource concentration levels (0.187, 0.367, 0.550, and 0.733 mg ml^–1^, respectively). For the E-L equation and the MPM, juvenile survival decreased at a fixed rate per time step. (**e – h**) $r_{\text{m}}$ TPCs at the same resource levels as (a – d) but for the E-L equation and the MPM, juvenile survival only decreased at the juvenile-to-adult transition point. Symbols denote mean $r_{\text{m}}$ ± standard deviation (error bars) calculated from the $r_{\text{m}}$ calculations for each treatment’s replicates (n=3; replicate-level $r_{\text{m}}$ calculations are provided in the manuscript’s accompanying GitHub repository). $r_{\text{m}}$ TPCs were fitted to Eqn SI1 using rTPC (Padfield et al., 2021). Asterisks (*) indicate TPCs (blue) fitted to MPM-derived $r_{\text{m}}$ calculations (triangles) using GAMs. Bootstrapping (residual resampling) was used to calculate 95% prediction bounds for each $r_{\text{m}}$ TPC. Negative $r_{\text{m}}$ calculations were cut off at –0.2 for plotting.

At all resource levels in the larval competition dataset (Huxley et al., 2022), the AE-L model and the E-L equation predicted $r_{\text{m}}$ to respond unimodally to temperature — it increased as temperatures increased from 22 to 32–34°C before declining rapidly to negative growth (figure 1). For both characterisations of juvenile survival, the $r_{\text{m}}$ calculations for the AE-L model and the E-L equation increased from ∼0.1 at 22°C to ∼0.2 at 34°C at resource levels above 0.183 mg ml^−1^. In contrast, the $r_{\text{m}}$ calculations from the AE-L model differed from the E-L equation derived $r_{\text{m}}$ calculations at 0.183 mg ml^−1^. The AE-L model predicted $r_{\text{m}}$ to increase from 0.07 at 22°C to 0.19 at 34°C, whereas the E-L equation predicted $r_{\text{m}}$ to increase from 0.02 to 0.13 across these temperatures. Under transitional decay at higher resource levels (>0.183 mg ml^−1^), the MPM-derived $r_{\text{m}}$ calculations were generally consistent with the $r_{\text{m}}$ calculations from the A-EL model and the E-L equation (figure 1e–h), but, at 0.183 mg ml^−1^, MPM-derived $r_{\text{m}}$ was only positive at 34°C (figure 1e).

At all resource levels, the MPM-derived $r_{\text{m}}$ calculations differed from the other approaches when juvenile survival decayed exponentially. Under this assumption at 0.183 mg ml^−1^, MPM-derived $r_{\text{m}}$ was negative at all temperatures, and, at higher resource levels MPM-derived $r_{\text{m}}$ was negative or close to zero at temperatures below 26°C. MPM-derived $r_{\text{m}}$ increased above this temperature, but it became negative again at 34°C or peaked at lower value than it did for the AE-L model and the E-L equation (figure 1c & d).

**Figure 2:**
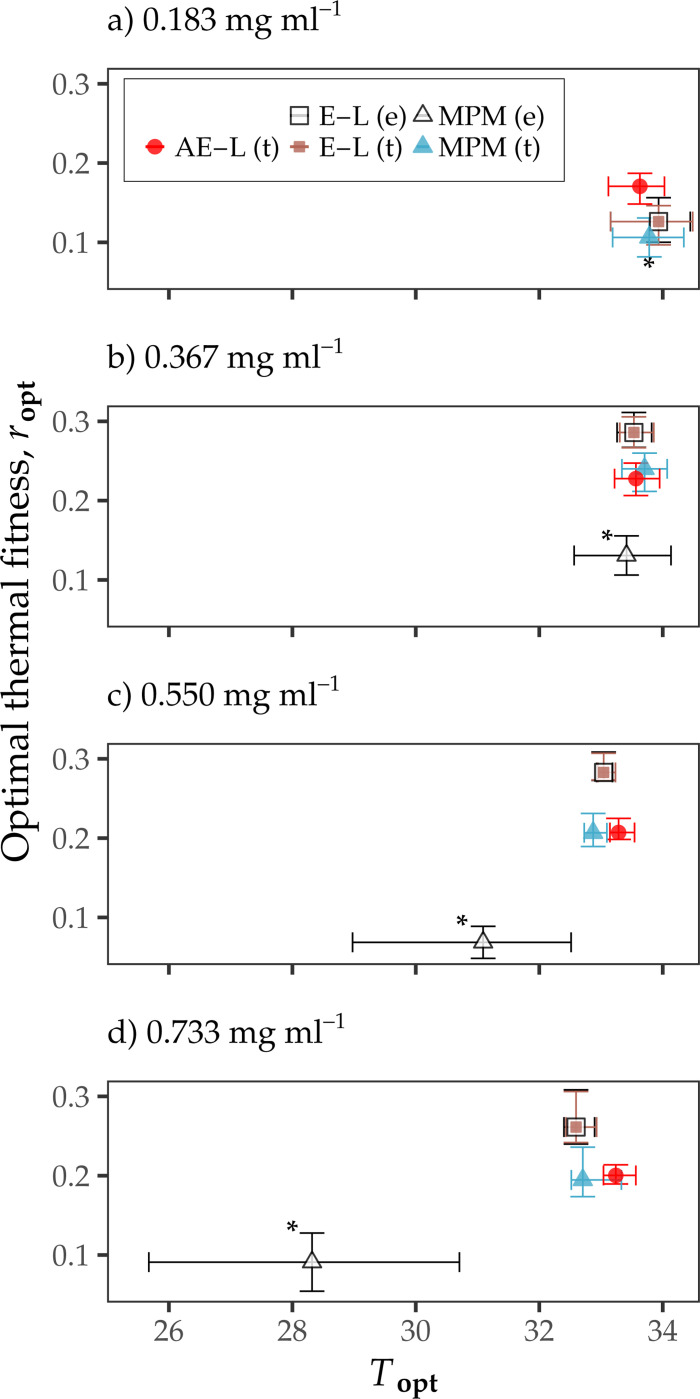
Comparison of predicted optimal thermal fitness ($r_{\text{opt}}$) at $T_{\text{opt}}$ for the analytic $r_{\text{m}}$ model (AE-L), the Euler-Lotka equation (E-L), and the MPM for the resource competition dataset (Huxley et al., 2022). **a-d** Predicted $r_{\text{opt}}$ at $T_{\text{opt}}$ for the three $r_{\text{m}}$ models across increasing larval resource concentration levels. Predictions (medians) with bi-directional error bars for when juvenile survival decreased at a fixed rate per time step are denoted “(e)” in the legend. Predictions for when juvenile survival only decreased at the juvenile-to-adult transition point are denoted “(t)”. Points with asterisks (*) are $r_{\text{opt}}\text{s}$ from TPCs fitted to MPM-derived $r_{\text{m}}$ calculations using GAMs. Bootstrapping (residual resampling) was used to calculate 95% uncertainty bounds for each point.

Except for the MPM TPC at 0.733 mg ml^−1^ under exponential decay, the TPCs for all three $r_{\text{m}}$ models predicted $r_{\text{m}}$ to peak ($T_{\text{opt}}$) between 31–34°C, irrespective of juvenile survival assumption or resource level (figure 2, table SI4). Optimal thermal fitness $\left(r_{\text{opt}}\right)$ was, in most cases, consistent across resource levels and survival assumptions for all $r_{\text{m}}$ models. It was not possible to predict $r_{\text{opt}}$ under exponential decay for the MPM because $r_{\text{m}}$ was negative at all temperatures. However, at 0.183 mg ml^−1^ under transitional decay, MPM $r_{\text{opt}}$ was predicted to be lower than $r_{\text{opt}}$ for both the AE-L model (0.11 compared to 0.17, respectively), and the E-L equation (0.13). At all resource levels under exponential decay, MPM $r_{\text{opt}}$ was predicted to be lower than MPM $r_{\text{opt}}$ under transitional decay. The $r_{\text{opt}}$ predictions for the MPM under exponential decay were also lower than the $r_{\text{opt}}$ predictions for the AE-L model and the E-L equation, as noted above (figure 2, table SI4).

### *Comparison of the* $r_{\text{m}}$ *calculations using the larval resource supply dataset*

**Figure 3:**
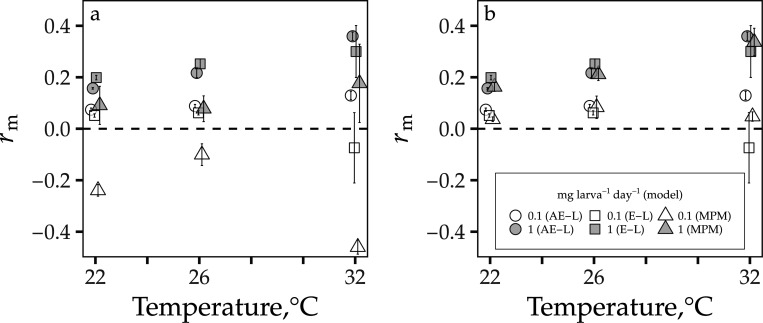
Comparison of $r_{\text{m}}$ calculations from discrete- and continuous-time $r_{\text{m}}$ models for the resource limitation dataset (Huxley et al., 2021). Estimated $r_{\text{m}}$ from the analytic $r_{\text{m}}$ model (AE-L), the Euler-Lotka equation (E-L), and the MPM at two larval resource supply levels (0.1 and 1 mg larva^–1^ day^–1^, white and grey symbols, respectively). (**a**) Calculations under the assumption that juvenile survival decreases at a fixed rate per time step. (**b**) Calculations when juvenile survival is assumed to only decrease at the juvenile-to-adult transition. Symbols denote mean $r_{\text{m}}$ ± standard deviation (error bars) calculated from the $r_{\text{m}}$ calculations for each treatment’s replicate populations (n=3; replicate-level $r_{\text{m}}$ calculations are provided in the manuscript’s accompanying GitHub repository.)

Under both survival assumptions at high-resource supply (1 mg larva^−1^ day^−1^) in the resource limitation dataset (Huxley et al., 2021), all $r_{\text{m}}$ models predicted $r_{\text{m}}$ to be positive and increase monotonically with temperature to its peak at 32°C. Under both juvenile survival assumptions at high-resource supply, differences between the AE-L and the E-L were small (figure 3ab, table SI6), but MPM $r_{\text{m}}$ under exponential decay was lower than $r_{\text{m}}$ from the AE-L model and the E-L (figure 3a, table SI6).

At low-resource supply (0.1 mg larva^−1^ day^−1^) under the exponential decay assumption in the larval resource supply dataset, the MPM $r_{\text{m}}$ calculations were always negative (figure 3a, table SI6). In contrast, AE-L model $r_{\text{m}}$ was always positive, and the E-L equation $r_{\text{m}}$ calculations were positive at 22 and 26°C before becoming negative at 32°C. The MPM predicted $r_{\text{m}}$ to increase from –0.24 at 22°C to –0.10 at 26°C; it then decreased to –0.46 at 32°C. The AE-L model predicted $r_{\text{m}}$ to increase with temperature from 0.07 at 22°C to 0.09 at 26°C, and 0.13 at 32°C, and the E-L equation predicted $r_{\text{m}}$ to increase from 0.05 at 22°C to 0.06 at 26°C and then decrease to –0.07 at 32°C.

## Discussion

Our goal was to examine the extent to which an analytic $r_{\text{m}}$ model based on the continuous E-L equation (the AE-L model; Eqn 4) can provide similar calculations of $r_{\text{m}}$ when parameterized with data across resource availability gradients, in comparison with that calculated from the (discrete) E-L equation (Eqn 2) and the MPM (Eqn 1).

Using two datasets that describe how resource availability and temperature jointly affect traits, and thus the predicted temperature dependence of $r_{\text{m}}$ (Huxley et al., 2021, 2022), we show that the AE-L model consistently yields similar calculations to the discrete time E-L equation when populations are not constrained by larval resource limitation or competition effects. This is as we expect, since the AE-L is based on the continuous time version of the E-L equation as we explain further below. We also show that modellers should proceed with caution when resource limitation or competition effects are expected to strongly mediate the temperature dependence of $r_{\text{m}}$ as model predictions differ in these cases.

For both datasets (Huxley et al., 2021, 2022), when resource availability was high and juvenile survival was assumed to only decrease at the juvenile-to-adult transition point, the AE-L model was also consistently in agreement with the MPM $r_{\text{m}}$ calculations across temperatures. Under transitional decay, the margin of error between all sets of $r_{\text{m}}$ calculations for the high resource treatments was generally small (figures 1f–h, 3b; tables SI2, SI3, SI6).

Under transitional decay at resource levels higher than 0.183 mg ml^−1^ in the larval competition dataset (Huxley et al., 2022), all approaches predicted $r_{\text{m}}$ to respond unimodally to temperature — increasing with temperature from 22 to ∼33°C, and then declining rapidly to zero after this peak (figures 1, 2; tables SI2–SI4). Similarly, for the high-resource supply (1 mg larva^−1^ day^−1^) in the resource limitation dataset (Huxley et al., 2021), the AE-L model consistently agreed with the $r_{\text{m}}$ calculations from the E-L equation and the MPM (i.e., again, the margin of error between the three sets of $r_{\text{m}}$ calculations for these treatments was generally not substantial; figure 3c, table SI6). For the high-resource supply treatments in the resource limitation dataset (Huxley et al., 2021) and for both survival assumptions, all approaches (with the exception of the small decrease in $r_{\text{m}}$ as temperatures increased from 22 to 26°C for the MPM) predicted $r_{\text{m}}$ to be positive and increase monotonically as temperatures increased from 22 to 32°C.

In contrast, for both datasets (Huxley et al., 2021, 2022) when resource availability was low, the AE-L model calculations and the MPM $r_{\text{m}}$ calculations markedly diverged (figures 1ab & 3a, tables SI2, SI3 & SI6). The largest discrepancy between the three approaches in the larval competition dataset (Huxley et al., 2022) was at the lowest resource level (0.183 mg ml^−1^ day^−1^) under the assumption that juvenile survival decreased by a fixed rate per time step. It was not possible to estimate $T_{\text{opt}}$ and $r_{\text{opt}}$ for the MPM under this assumption because $r_{\text{m}}$ was negative across all of the temperature range, yet both the AE-L model and the E-L equation predicted $T_{\text{opt}}$ to be close to 34°C and $r_{\text{opt}}$ to be ∼0.15. There were also important differences between the $r_{\text{m}}$ models for the low resource level under transitional decay of juvenile survival. The three approaches predicted $T_{\text{opt}}$ to be close to 34°C and $r_{\text{opt}}$ to be ∼0.15, but the E-L equation predicted $r_{\text{m}}$ to be close to zero between 22 and 32°C, and the MPM differed in its prediction of the sign of $r_{\text{m}}$ across much of the temperature range (figure 1e, table SI4).

Under the model assumption of exponentially decaying juvenile survival, an even greater level of disparity was observed between the AE-L prediction and the MPM model for the resource limitation dataset (Huxley et al., 2021) at low-resource supply (0.1 mg larva^−1^ day^−1^). In this case, the AE-L model predicted $r_{\text{m}}$ to be positive (and increasing) across the entire temperature range, whereas as the MPM predicted $r_{\text{m}}$ to be negative across this range. The E-L $r_{\text{m}}$ prediction was intermediate between them (figure 3a, table SI6).

This disagreement in the calculations of $r_{\text{m}}$ amongst the models at low resource levels (Huxley et al., 2021, 2022) stems from differences in their mathematical structure. Stage-structured MPMs project population abundance over discrete time intervals, assume discrete stages, and (as formulated here) explicitly consider all juvenile stages. In contrast, the continuous-time AE-L model aggregates these stages into a single, continuous stage described by a single, cumulative juvenile mortality and development time parameter (zJ and α, respectively). This simplifying assumption of the AE-L model appears to affect the calculations of $r_{\text{m}}$ when populations are exposed to environmental conditions that cause delays in the system.

Also, any differences between the $r_{\text{m}}$ models must stem from the aggregation of juvenile mortality and development in the AE-L model because we have essentially compared $r_{\text{m}}$ calculated from the discrete-time Euler-Lotka equation (Eqn 2) to an approximation of $r_{\text{m}}$ derived from the continuous Euler-Lotka equation (Eqn 3). Essentially, the aggregation of juvenile mortality and development in the AE-L model makes it relatively insensitive to juvenile survival. While the discrete-time E-L equation is more sensitive to proportional juvenile survival at transition, the fact that its $r_{\text{m}}$ calculations were the same irrespective of how survival decreased prior to the juvenile-to-adult transition point shows that it is insensitive to the specifics of how juvenile survival is characterised. The MPM, on the other hand, is highly sensitive (perhaps too sensitive) to how juvenile survival is characterised. This key finding shows that standard practices for characterising juvenile survival, whether that be integrating over time steps (in the case of the AE-L) or assuming a constant rate of mortality (i.e., MPMs) are problematic, particularly when resources are limiting, and as a result may be age or size specific. Indeed, different juvenile survival characterisations can also introduce substantial bias when converting age-structured vital rates estimated from life tables to calculate $r_{\text{m}}$ using MPMs that are only stage-structured (Fujiwara and Diaz-Lopez, 2017; Kendall et al., 2019).

Environmental differences that cause time delays in life history can be directly accounted for in MPMs by increasing the number of columns in the transition matrix (M, Eqn 1) assigned to a particular life stage. For example, intensified larval competition at low resource levels increases development time across all juvenile stages (Huxley et al., 2022). This effect can be accounted for in MPMs by increasing the number of columns in M assigned to the juvenile life stages. In this way, MPMs implicitly include time delays. This implicit “stretching” of M to account for delays makes it possible to study how environment-driven delays affect $r_{\text{m}}$. Delay effects can also be studied using continuous-time stage structured population models (Gurney et al., 1983; Nisbet and Gurney, 1983) that introduce delays explicitly with delay parameters (thus yielding delay-differential equations). In contrast, by merging the juvenile stages into a single, continuous stage, the AE-L model may not adequately weight the negative impact that delay mechanisms can have on $r_{\text{m}}$ since these factors are effectively summarized in a single parameter representing the expected time of maturation. This simplifying assumption of the analytic $r_{\text{m}}$ model implies that delay mechanisms are expected to only have negligible effects on $r_{\text{m}}$.

The results of the MPM sensitivity analyses in Huxley et al. (2021, 2022) are qualitatively similar with the sensitivity analysis of the AE-L model in Cator et al. (2020) in showing that juvenile traits contribute more to $r_{\text{m}}$ than adult traits. This also supports the notion that the AE-L may not reliably estimate $r_{\text{m}}$ in low resource conditions because it does not adequately account for the negative effect of increased juvenile mortality on $r_{\text{m}}$. Additional examination of these sensitivity analyses provides further insights into the behavior of the AE-L model’s estimate of $r_{\text{m}}$ in low resource conditions. For example, the sensitivity analysis for the AE-L model indicates that juvenile survival contributes a smaller proportion to $r_{\text{m}}$ than indicated by the MPM sensitivity analysis and this model’s $r_{\text{m}}$ calculations shown here. While the MPM sensitivity analyses in Huxley et al. (2021, 2022) indicate that together juvenile development time and survival contribute more to $r_{\text{m}}$ than adult traits do, their respective contributions $r_{\text{m}}$ cannot be easily separated. However, when the MPM sensitivity analyses in Huxley et al. (2021, 2022) are used in combination with the MPM $r_{\text{m}}$ calculations reported here at low resource levels, it is clear that MPM $r_{\text{m}}$ is more sensitive to juvenile survival than development time. For example, the number of matrix columns assigned to the juvenile stages (i.e., development time) were identical for the low resource supply level MPMs in Huxley et al. (2021), yet under exponentially decaying survival the MPM predicted $r_{\text{m}}$ to be –0.24 compared to 0.04 under transitional decay.

The first implication of this finding is that not all of the formulations of the intrinsic growth rate of a population may be equally accurate for all types of organisms in all situations. As we find here, for organisms where juvenile survival patterns are neither constant nor exponentially distributed (either generally or due to environmental conditions) the choice of metric matters to conclusions drawn about the population. Thus either an MPM approach may be preferred (when a discretization is appropriate) or when a continuous time model is preferred, a recognition that the calculated $r_{\text{m}}$ may be overly optimistic should be kept in mind. Although the analytic AE-L model may be unreliable at low resource levels, a key finding of this study — that the AE-L model can reliably predict $r_{\text{m}}$ when populations are not constrained by resource limitation or larval competition effects — has important implications for the efficiency of study workflows. For example, this method allows for much easier integration of laboratory measures of trait performance to investigate the effect of temperature at high resource supply than individually constructing MPMs for each treatment. Furthermore, with respect to VBD transmission frameworks specifically, this result shows that continuous-time analytic $r_{\text{m}}$ models may offer a simple method for “plugging in” $r_{\text{m}}$ responses into continuous-time VBD models. Integrating these models into broader VBD model frameworks could improve their reliability in predicting of how temperature and high resource levels together affect transmission risk through their effects on vector $r_{\text{m}}$.

The second important implication of this study’s central finding relates to whether existing mathematical models can be used to predict and understand the population-level effects of environmental change on temperature-sensitive organisms that have complex life histories. The answer to this question lies with two connected ideas. First, whether under temperature fluctuations alone any of these approaches capture meaningful summaries of population performance either in aggregate or instantaneously. Currently, calculations of $r_{\text{m}}$ at fixed temperatures are often averaged or used for rate summation-type approaches in order to estimate a realized/time-averaged $r_{\text{m}}$. However, we know of no laboratory experiments that confirm multi-generational population growth rates under known fluctuation regimes are well captured by the approach (although in other contexts the generalization from constant to varying temperature is known to be fraught). Further, whether these metrics are accurate (or useful) measures of temperature-dependent $r_{\text{m}}$ in the field and how they are affected by other environmental factors, such as resource availability must be assessed. For example, existing model frameworks (e.g., continuous-time stage-structured population models; Amarasekare and Coutinho, 2013; Beck-Johnson et al., 2013) can be developed to incorporate temperature- and resource-induced developmental delays, but datasets that describe these combined effects in the field are largely absent. The absence of such datasets is probably due to the fact that new measures are needed to determine how effective temperature-dependent $r_{\text{m}}$ in the field is affected by resource fluctuations. Further, realistic and tractable measures of density-dependent effects on $r_{\text{m}}$ are needed to be able for predicting the effects of environmental change on insect abundance dynamics, in general.

Semi-field systems could provide opportunities to track the entire insect life cycle under ambient environmental conditions. For example, in *Drosophila*, such systems have recently been used to observe thermal adaptation in response to natural environmental change by tracking the evolution of fitness-associated phenotypes and allele frequencies (Rudman et al., 2022) across generations. In mosquitoes, such systems have generally been used to test the effectiveness of novel bio-control strategies, such as transgenic fungi (Lovett et al., 2019), but they also could allow for the effects of temperature × resource interactions in the larval stage on fitness and abundance to be explored under conditions which more closely resemble natural environments. Further insights could be provided by examining the interaction between insects and microbes, for example. Recent studies show that mosquitoes can be reared exclusively on cultures of *Asaia* bacteria (Chouaia et al., 2012; Souza et al., 2019), while other studies have shown that larval exposure to microbial variability can affect adult mosquito life history traits (Dickson et al., 2017). However, microbiota at mosquito breeding sites is spatially heterogeneous (Hery et al., 2021), which could mean that any generalizable patterns in resource availability could be difficult to detect.

In spite of the difficulties posed by this challenge, greater research effort towards this important issue is needed, especially if the goal is to understand how insects, including vector populations and VBD transmission patterns, will respond to climatic warming. Indeed, resource availability itself is likely to be temperature-dependent because microbial growth rates also increase with temperature (Craine et al., 2010; Cross et al., 2015; Smith et al., 2019). In this way, increases in environmental temperatures could increase the concentration of food in the environment, which will increase population growth through decreased juvenile development time and increased adult recruitment rates. This could contribute to the expansion of disease vectors and other invasive insect species into regions that were previously prohibitive by broadening $r_{\text{m}}’\text{s}$ thermal niche width (Amarasekare and Simon, 2020; Huxley et al., 2021, 2022; Lehmann et al., 2020).

Recent studies have used $r_{\text{m}}$ calculations from the Euler-Lotka equation to predict population viability under climatic warming (Deutsch et al., 2008; Tewksbury et al., 2008), but very few studies have assessed whether it can provide reliable estimates of $r_{\text{m}}$ when populations are exposed to multiple environmental factors. Our study shows that analytic models based on the Euler-Lotka equation can provide similar calculations of temperature-dependent $r_{\text{m}}$ to standard methods providing resource environments are non-limiting. This study also underlines the need for accurate measures of how variation in resource availability in the field can affect the thermal response of $r_{\text{m}}$ and therefore, abundance. Such data are particularly key to improving predictions of how climatic warming will affect seasonal insect populations through its effects on abundance dynamics.

## Supplementary Material

1

## Data Availability

All data are in the fully open VecTraits database. All data and code to reproduce the results, figures and tables are in an anonymised Github repository.

## References

[R1] Amarasekare P., and Coutinho R. M.. 2013. The intrinsic growth rate as a predictor of population viability under climate warming. Journal of Animal Ecology 82:1240–1253. 23926903 10.1111/1365-2656.12112

[R2] Amarasekare P., and Savage V.. 2012. A framework for elucidating the temperature dependence of fitness. The American Naturalist 179:178–191. 10.1086/663677 22218308

[R3] Amarasekare P., and Simon M. W.. 2020. Latitudinal directionality in ectotherm invasion success. Proceedings of the Royal Society B 287:20191411. 32075530 10.1098/rspb.2019.1411 PMC7031664

[R4] Bar-On Y. M., Phillips R., and Milo R.. 2018. The biomass distribution on earth. Proceedings of the National Academy of Sciences 115:6506–6511. 10.1073/pnas.1711842115 PMC6016768 29784790

[R5] Beck-Johnson L. M., Nelson W. A., Paaijmans K. P., Read A. F., Thomas M. B., and Bjørnstad O. N.. 2013. The effect of temperature on anopheles mosquito population dynamics and the potential for malaria transmission. PLOS one 8:e79276. 24244467 10.1371/journal.pone.0079276 PMC3828393

[R6] Birch L. 1948. The intrinsic rate of natural increase of an insect population. The Journal of Animal Ecology 17:15–26.

[R7] Branda F., Nakase T., Maruotti A., Scarpa F., Ciccozzi A., Romano C., Peletto S., Bispo de Filippis A. M., Alcantara L. C., Marcello A., 2023. Dengue virus transmission in italy: surveillance and epidemiological trends up to 2023. medRxiv pages 2023–12. 10.1038/s41597-024-04162-7 PMC11621350 39639007

[R8] Brass D. P., Cobbold C. A., Ewing D. A., Purse B. V., Callaghan A., and White S. M.. 2021. Phenotypic plasticity as a cause and consequence of population dynamics. Ecology Letters 24:2406–2417. 34412157 10.1111/ele.13862

[R9] Caswell H. 1989. Matrix population models: Construction, vol. 255. Sinauer Associates.

[R10] Cator L. J., Johnson L. R., Mordecai E. A., El Moustaid F., Smallwood T. R., LaDeau S. L., Johansson M. A., Hudson P. J., Boots M., Thomas M. B., 2020. The role of vector trait variation in vector-borne disease dynamics. Frontiers in ecology and evolution 8:189. 32775339 10.3389/fevo.2020.00189 PMC7409824

[R11] Chouaia B., Rossi P., Epis S., Mosca M., Ricci I., Damiani C., Ulissi U., Crotti E., Daffonchio D., Bandi C., 2012. Delayed larval development in anopheles mosquitoes deprived of asaia bacterial symbionts. BMC microbiology 12:1–8. 22375964 10.1186/1471-2180-12-S1-S2 PMC3287513

[R12] Cole L. C. 1954. The population consequences of life history phenomena. The Quarterly review of biology 29:103–137. 13177850 10.1086/400074

[R13] Costanza R., d’Arge R., De Groot R., Farber S., Grasso M., Hannon B., Limburg K., Naeem S., O’neill R. V., Paruelo J., 1997. The value of the world’s ecosystem services and natural capital. nature 387:253–260.

[R14] Craine J. M., Fierer N., and McLauchlan K. K.. 2010. Widespread coupling between the rate and temperature sensitivity of organic matter decay. Nature Geoscience 3:854–857.

[R15] Cross W. F., Hood J. M., Benstead J. P., Huryn A. D., and Nelson D.. 2015. Interactions between temperature and nutrients across levels of ecological organization. Global change biology 21:1025–1040. 25400273 10.1111/gcb.12809

[R16] Deutsch C. A., Tewksbury J. J., Huey R. B., Sheldon K. S., Ghalambor C. K., Haak D. C., and Martin P. R.. 2008. Impacts of climate warming on terrestrial ectotherms across latitude. Proceedings of the National Academy of Sciences 105:6668–6672. 10.1073/pnas.0709472105 PMC2373333 18458348

[R17] Dickson L. B., Jiolle D., Minard G., Moltini-Conclois I., Volant S., Ghozlane A., Bouchier C., Ayala D., Paupy C. V. Moro, 2017. Carryover effects of larval exposure to different environmental bacteria drive adult trait variation in a mosquito vector. Science advances 3:e1700585. 28835919 10.1126/sciadv.1700585 PMC5559213

[R18] Ellner S. P., and Rees M.. 2006. Integral projection models for species with complex demography. The American Naturalist 167:410–428. 10.1086/499438 16673349

[R19] Frazier M., Huey R. B., and Berrigan D.. 2006. Thermodynamics constrains the evolution of insect population growth rates:“warmer is better”. The American Naturalist 168:512–520. 10.1086/506977 17004222

[R20] Fujiwara M., and Diaz-Lopez J.. 2017. Constructing stage-structured matrix population models from life tables: comparison of methods. PeerJ 5:e3971. 29085763 10.7717/peerj.3971 PMC5660883

[R21] Gurney W., Nisbet R., and Lawton J.. 1983. The systematic formulation of tractable single-species population models incorporating age structure. The Journal of Animal Ecology 52:479–495.

[R22] Hery L., Guidez A., Durand A.-A., Delannay C., Normandeau-Guimond J., Reynaud Y., Issaly J., Goindin D., Legrave G., Gustave J., 2021. Natural variation in physicochemical profiles and bacterial communities associated with aedes aegypti breeding sites and larvae on guadeloupe and french guiana. Microbial ecology 81:93–109. 32621210 10.1007/s00248-020-01544-3 PMC7794107

[R23] Høye T. T., Loboda S., Koltz A. M., Gillespie M. A., Bowden J. J., and Schmidt N. M.. 2021. Non-linear trends in abundance and diversity and complex responses to climate change in arctic arthropods. Proceedings of the National Academy of Sciences 118:e2002557117. 10.1073/pnas.2002557117 PMC7812779 33431570

[R24] Huey R. B., and Berrigan D.. 2001. Temperature, demography, and ectotherm fitness. The American Naturalist 158:204–210. 10.1086/321314 18707349

[R25] Huxley P. J., Murray K. A., Pawar S., and Cator L. J.. 2021. The effect of resource limitation on the temperature dependence of mosquito population fitness. Proc. R. Soc. B Biol. Sci. 288:rspb.2020.3217. 10.1098/rspb.2020.3217 PMC8079993 33906411

[R26] Huxley P. J., Murray K. A., Pawar S., and Cator L. J.. 2022. Competition and resource depletion shape the thermal response of population fitness in Aedes aegypti. Communications Biology 5:1–11. 35046515 10.1038/s42003-022-03030-7 PMC8770499

[R27] Kendall B. E., Fujiwara M., Diaz-Lopez J., Schneider S., Voigt J., and Wiesner S.. 2019. Persistent problems in the construction of matrix population models. Ecological modelling 406:33–43.

[R28] Kontopoulos D.-G., Garcıa-Carreras B., Sal S., Smith T. P., and Pawar S.. 2018. Use and misuse of temperature normalisation in meta-analyses of thermal responses of biological traits. PeerJ 6:e4363. 29441242 10.7717/peerj.4363 PMC5808315

[R29] Lawlor J. A., Comte L., Grenouillet G., Lenoir J., Baecher J. A., Bandara R., Bertrand R., Chen I.-C., Diamond S. E., Lancaster L. T., 2024. Mechanisms, detection and impacts of species redistributions under climate change. Nature Reviews Earth & Environment pages 1–18.

[R30] Lehmann P., Ammunét T., Barton M., Battisti A., Eigenbrode S. D., Jepsen J. U., Kalinkat G., Neuvonen S., Niemelä P., Terblanche J. S., 2020. Complex responses of global insect pests to climate warming. Frontiers in Ecology and the Environment 18:141–150.

[R31] Lotka A. J. 1907. Relation between birth rates and death rates. Science 26:21–22. 10.1126/science.26.653.21-a 17754777

[R32] Lovett B., Bilgo E., Millogo S. A., Ouattarra A. K., Sare I., Gnambani E. J., Dabire R. K., Diabate A., and St. Leger R. J.. 2019. Transgenic metarhizium rapidly kills mosquitoes in a malaria-endemic region of burkina faso. Science 364:894–897. 31147521 10.1126/science.aaw8737

[R33] Nakase T., Giovanetti M., Obolski U., and Lourenço J.. 2023. Global transmission suitability maps for dengue virus transmitted by aedes aegypti from 1981 to 2019. Scientific Data 10:275. 37173303 10.1038/s41597-023-02170-7 PMC10182074

[R34] Nisbet R., and Gurney W.. 1983. The systematic formulation of population models for insects with dynamically varying instar duration. Theoretical Population Biology 23:114–135.

[R35] Oliveira S., Rocha J., Sousa C. A., and Capinha C.. 2021. Wide and increasing suitability for aedes albopictus in europe is congruent across distribution models. Scientific reports 11:9916. 33972597 10.1038/s41598-021-89096-5 PMC8110805

[R36] Outhwaite C. L., McCann P., and Newbold T.. 2022. Agriculture and climate change are reshaping insect biodiversity worldwide. Nature 605:97–102. 35444282 10.1038/s41586-022-04644-x

[R37] Padfield D., O’Sullivan H., and Pawar S.. 2021. rtpc and nls. multstart: a new pipeline to fit thermal performance curves in r. Methods in Ecology and Evolution 12:1138–1143.

[R38] Pawar S., Huxley P. J., Smallwood T. R., Nesbit M. L., Chan A. H., Shocket M. S., Johnson L. R., Kontopoulos D.-G., and Cator L. J.. 2024. Variation in temperature of peak trait performance constrains adaptation of arthropod populations to climatic warming. Nature Ecology & Evolution pages 1–11. 38273123 10.1038/s41559-023-02301-8 PMC10927549

[R39] Pecl G. T., Araújo M. B., Bell J. D., Blanchard J., Bonebrake T. C., Chen I.-C., Clark T. D., Colwell R. K., Danielsen F., Evengård B., 2017. Biodiversity redistribution under climate change: Impacts on ecosystems and human well-being. Science 355:eaai9214. 28360268 10.1126/science.aai9214

[R40] R Core Team. 2023. R: A Language and Environment for Statistical Computing. R Foundation for Statistical Computing, Vienna, Austria.

[R41] Rudman S. M., Greenblum S. I., Rajpurohit S., Betancourt N. J., Hanna J., Tilk S., Yokoyama T., Petrov D. A., and Schmidt P.. 2022. Direct observation of adaptive tracking on ecological time scales in drosophila. Science 375:eabj7484. 35298245 10.1126/science.abj7484 PMC10684103

[R42] Ryan S. J., Carlson C. J., Tesla B., Bonds M. H., Ngonghala C. N., Mordecai E. A., Johnson L. R., and Murdock C. C.. 2021. Warming temperatures could expose more than 1.3 billion new people to zika virus risk by 2050. Global Change Biology 27:84–93. 33037740 10.1111/gcb.15384 PMC7756632

[R43] Sánchez-Bayo F., and Wyckhuys K. A.. 2019. Worldwide decline of the entomofauna: A review of its drivers. Biological conservation 232:8–27.

[R44] Schoolfield R. M., Sharpe P., and Magnuson C. E.. 1981. Non-linear regression of biological temperature-dependent rate models based on absolute reaction-rate theory. Journal of theoretical biology 88:719–731. 6790878 10.1016/0022-5193(81)90246-0

[R45] Skendžić S., Zovko M., Ž ivković I. P., Lešić V., and Lemić D.. 2021. The impact of climate change on agricultural insect pests. Insects 12:440. 34066138 10.3390/insects12050440 PMC8150874

[R46] Smith T. P., Thomas T. J., Garćıa-Carreras B., Sal S., Yvon-Durocher G., Bell T., and Pawar S.. 2019. Community-level respiration of prokaryotic microbes may rise with global warming. Nature communications 10:1–11. 10.1038/s41467-019-13109-1 PMC6851113 31719536

[R47] Souza R. S., Virginio F., Riback T. I. S., Suesdek L., Barufi J. B., and Genta F. A.. 2019. Microorganism-based larval diets affect mosquito development, size and nutritional reserves in the yellow fever mosquito aedes aegypti (diptera: Culicidae). Frontiers in physiology 10:152. 31024326 10.3389/fphys.2019.00152 PMC6465640

[R48] Stubben C. J., and Milligan B. G.. 2007. Estimating and analyzing demographic models using the popbio package in r. Journal of Statistical Software 22.

[R49] Tewksbury J., Huey R., and Deutsch C.. 2008. Climate warming puts the heat on tropical ectotherms. Science 320:1296–1297. 18535231 10.1126/science.1159328

[R50] Winsor C. P. 1932. The gompertz curve as a growth curve. Proceedings of the national academy of sciences 18:1–8. 10.1073/pnas.18.1.1 PMC1076153 16577417

[R51] Wood S., and Wood M. S.. 2015. Package ‘mgcv’. R package version 1:729.

[R52] Wood S. N. 2011. Fast stable restricted maximum likelihood and marginal likelihood estimation of semiparametric generalized linear models. Journal of the Royal Statistical Society Series B: Statistical Methodology 73:3–36.

