# Supplementary material for: Divergence of discrete- versus continuous-time calculations of the temperature dependence of maximum population growth rate
Source: Res Sq. 2024 Nov 14:rs.3.rs-5361425. Preprint. [Version 1] doi: 10.21203/rs.3.rs-5361425/v1 (PMC11601820; doi:10.21203/rs.3.rs-5361425/v1)
Supplement: 1 [file NIHPPRS5361425v1-supplement-1.pdf]

Supplementary Files

This is a list of supplementary files associated with this preprint. Click to download.

- NatMethodssupportinginformation.pdf
